# Environmental safety evaluation of geopark based on CPTED concept and fuzzy comprehensive analysis

**DOI:** 10.1371/journal.pone.0260316

**Published:** 2021-11-22

**Authors:** Guoyi Chen, Shangmin Zhang, Bangquan Yan, Shengzhen Miao

**Affiliations:** 1 School of Business Management, Chongqing Three Gorges University, Chongqing, China; 2 Chongqing University of Arts and Sciences, Chongqing, China; Northeast Electric Power University, CHINA

## Abstract

In recent years, with the increasingly popular and openness of Geoparks, Environmental safety has become a major concern for sustainable geo-tourism. It is therefore necessary to conduct an environmental safety performance evaluation for promoting geo-tourism development. In order to identify and figure out the factors influencing the tourists’ environmental safety perception, an index system was established based on six principles of Crime Prevention Through environment design (CPTED) theory. A Questionnaire was adopted for data collection, and the overall evaluation value and concrete index scores at all levels were obtained via the fuzzy comprehensive analysis and Importance-Performance analysis. Empirical results show that: (1) tourists’ perception of environmental safety performance in Shilin Park from high to low was: image and maintenance, Natural Surveillance, territoriality, Access control, Activity support and target hardening; (2) The sub-factors influencing tourists’ safety perception mostly include electronic monitoring device, Lighting system, Public safety management, Road layout, environmental sanitation; While attention should be paid on the following aspects including park service center, inter-personal surveillance, surrounding environment, unobstructed view, parking lot, Signpost, for they are considered as high-importance items with relatively poor performance. Based on the analysis, three optimization measures were proposed, including optimizing the layout and design of each space, strengthening the deterrent force of the park and maintaining a good environmental image. This research provides useful suggestions for Geopark decision-makers on determining the priority of Geopark spatial planning and management, as well as achieving the optimal allocation of resources to promote the sustainable development of Geopark.

## Introduction

A Geopark is a nationally protected area that contains series of geological heritage sites with particularly important archaeological, ecological, historical [1], and cultural values [2]. A Geopark realizes its value mainly through a three-pronged approach, that is, Conservation, education and tourism [1–3]. Firstly, Geopark plays an important role to conserve the significant geological heritage sites [4]. Secondly, Geopark acts as a channel for communicating geo-scientific knowledge and environmental conservation concept to the public and society [5], thirdly it also stimulates the local economic development through geo-tourism and geo-sightseeing [6], and provides the opportunities of employment for local peasant [7]. According to [6–8], A Geopark, as an important part of the earth’s ecosystem, is also an integrated platform for urban residents’ sightseeing tourism, excursion, recreation, health-care, science and education, culture and education [1].

In China, with the rapid development of the economy and society, people’s demands for tourism destination such as Geoparks are increasingly gradually. On the one hand, Geoparks are the important places for tourists’ excursion, recreation and Geological heritage education. It has played an irreplaceable role in beautifying the living environment and maintaining the stability of the urban ecosystem [6, 9]. On the other hand, with the increasing openness of Geoparks and tightly linked with urban space, the frequency of unsafe incidents also increased [10]. Due to the feature of wide coverage and far away from the city center, the safety incidents of tourist occur frequently nowadays. These incidents remind us that it is necessary to establish an environmental safety evaluation index system and optimize geopark spatial planning from the perspective of tourists’ safety perception [11, 12]. However, in the academic circle, previously researchers mainly focus on the aesthetics and geological heritages of Geoparks, while neglecting the research about tourists’ perception of environmental safety.

To date, many studies have conducted environmental safety audit through CPTED, and most previous researches addressed safety design at the neighborhood level [13], Leisure center [14], central business centers [15], and transportation hubs [16]. Nonetheless, up to now, a dearth of research exists on environmental design and its influence on tourists’ safety perception in tourist attractions such as Geoparks. Although several scholars have begun to investigate the relationship between parks and Crime Prevention through Environmental Design (CPTED), and stated that reasonable design and properly planning of physical space could indeed decrease the occurrence of unsafe events and reduce tourists’ perception of fearless [17], they mainly focused on urban park while neglected Geoparks. Moreover, among the studies which have highlighted the importance of environmental design in park, most of them are experimental surveys carried out in western countries, however the research in China was seldom.

Furthermore, according to [18–20], every single factor, such as location, lighting and plants configuration, have associated with tourists’ perception of safety, however, these factors have not been systematically discussed and integrated from the perspective of spatial elements as a whole [21–23]. Therefore, there are still relatively limited researches that have been conducted to investigate Geopark environmental safety through Crime Prevention through Environmental Design (CPTED) principles. Thus, from this point of view, this is of significant theoretical value for establishing Geopark environmental safety assessment index based on CPTED.

This study provides insight into geopark environmental design and safety management for Geopark managers and holidaymakers. Through effective adoption of CPTED approaches, it is hoped that the findings will enhance physical and environmental design of the national geopark. Besides this, this study is expected to contribute to academic literature on the less-considered area of geopark in China. Thus, the research of environmental safety perception is of both academic and practical value for Geopark professionals.

Therefore, based on the theoretical framework of CPTED, the author combines the fuzzy comprehensive evaluation method with Importance-Performance analysis method to construct the Geopark environmental safety evaluation system from the perspective of tourist’s psychology perception, and takes Shilin Geopark as an example to validate the reliability and accuracy of this evaluation system. Finally, some reasonable suggestions and implications are put forward for environmental safety improvement.

## Theoretical foundation

### CPTED and tourist sites

First introduced by Jeffery (1971), CPTED theory stated that natural environment could provide opportunities for occurrence of crime [24], in other words, the reduction of crime incidences can be achieved by varying environmental factors. CPTED is defined as “the proper design and effective use of the built environment which can lead to a reduction in incidences of crime, and to an improvement in the quality of life” [25]. CPTED has also been considered as one of the most cost-effective crime prevention measures, for it reduced the overall cost of preventing crime through pre-design and plan of the environmental factors. Modern CPTED focuses on six main constructs: territoriality, Natural Surveillance, Access control, Activity support, image maintenance and target hardening, these six elements are mutually interrelated and interacted, and forming the main designing approaches of CPTED.

Territoriality refers to designing spaces in a way clearly distinguishing the private space from the public space or the semi-public space, and providing the users sense of ownership and proprietary [26], and then it is easy and obviously to identify the strangers and intruders [27]. Natural Surveillance signifies the ability to observe what happened in this area. It has directly influence on tourists’ perception of safety while using such space [28]. It can increase the visibility of the crime targets and reduce the probability of being violating. High level of Natural Surveillance means that people can see what other people are doing and therefore preventing some ’would-be’ criminal behavior [29]. Measure for Surveillance including unobstructed view, security Guard patrol, and electronic monitoring device (such as Closed-Circuit Television). In addition, the adoption of Bright lighting and interpersonal Surveillance also promoting the opportunities for surveillance [30].

Access control refers to the entrance permission to certain areas. It reduces opportunities for crime through setting access permissions in the main road and entrance design [31]. Measures for enhancing access control includes access control system, fencing, Signpost and walls [32].

Activity support means conducting series of legitimate activities in these areas to enhance interpersonal communication and thus reduce Incidence of crime [29, 31]. Through thoughtful design of geopark space together with conduction of interesting events, the tourists will be attracted to these areas. As [32] stated, criminals would be less likely to offend in the areas with higher level of “eyes on the street”, for their illegal activities may be seen and monitored to the most extent.

The concept of image maintenance originated from Wilson and Kelling’s Broken Window Theory [33]. In their view, if the broken windows of a building were not repaired timely, more windows would be broken deliberately, and someone even would break into the building [33]. Thus, from this point of view, the equipment and main roads should be maintained, and the rubbish should be cleaned regularly, then the image of well-organized and civilization would be perceived by a potential offender. Then their crime intention will be reduced. Furthermore [13], argued that environmental sanitation, Public facilities, Lighting system and Civilized behavior all contributed to the reduction of crime probability.

Target hardening means increasing difficulties for Committing a crime, measures includes adopting strong gate and windows, installing electronical alarms and so on. In tourists’ attractions, the installments of Safety bulletin boards, high-level of Geopark service and parking lot management, these all proved to be positively related with crime reduction.

Up to now, environmental design has been considered as one of the most important measures for enhancing tourists’ perception of safety in tourist attractions [34–36]. Based on the research of [34], high, thick and dense vegetation was proved to be positively related with crime incidences, and the figure will increase obviously once it was not carefully maintained [35]. In addition, legitimate activities conducted either within tourist attractions or surroundings were considered as an important factor for enhancing tourists’ perception of safety [14, 36].

Subsequently, some studies have also surveyed the relationship between Physical boundaries, electronic monitoring device and crime in urban tourism attractions [37]. Physical boundaries, such as the walls and plants between two areas may limit the possibilities for potential victims for escape, this then may increase tourists’ concern about safety. From this perspective of view, properly design of the enclosures, fences and road layouts is necessary for meeting tourists’ demand for privacy protection as well as Emergency escape passage [38]. Similarly [39], also reflected that the long-view distance road design and the easily access to nearby streets were also effectively ways for promoting tourists’ safety confidence.

It is worth mentioning that in some cases, the clean environment and equipment maintenance may provide tourists feeling of securities and also brought them the impression of high-level inner management [40]. Furthermore [14], has stated that poor lighting may lead to increasingly crime incidence in eco-tourism sites. In their work [14], found that bright lighting provides conditions for tourists’ Omnidirectional observation and acts timely to potential threats. Consistent with above [38], had added that some security precautions such as electronic monitoring device, Security guards patrol, public safety facilities and Safety bulletin board are also important means for crime prevention in eco-tourism attractions.

### Fuzzy IPA analysis

First introduced by [41], Importance and performance analysis (IPA) was been widely applied to investigate the critical attributes in market survey of customer satisfaction and loyalty. [42] argued that IPA is an effective method for identifying priorities of different attributes and directing different countermeasures. Researchers apply IPA to identify two dimensions of attributes: importance-level ones and performance-level ones. These two dimensions are then integrated into a matrix vividly. Drawing on this, researchers can easily figure out the primary driving factors of tourist satisfaction, and in subsequently adopt effectively countermeasures [43]. Therefore, IPA was considered as a useful method of resource allocation optimization under the context of limited resource.

On the other hand, tourists’ perception of safety is characterized by uncertainty and ambiguity, thus using traditional evaluation method which adopting Likert scale (equal-space crisp number) to define tourists’ subjective perception based on linguistic assessments becomes unfeasible [44]. The reason underlying it is that individual perceptions and feelings are subjective and fuzzy, even the same describing words may represent various meanings. Therefore, the use of crisp numbers to describe human feelings or perception is not feasible. In 1965, Zadeh noted that fuzzy theory can deal with problems involving uncertainty and ambiguity, and fuzzy number is more suitable than crisp number for analyzing linguistic term scale about tourists’ perception of hospitality service. As stated by [45], the advantages of modeling by fuzzy number lies in that it described and evaluated personal feelings and attitude naturally. Thus, fuzzy comprehensive analysis is needed for psychometrically measurement of tourists’ attitude performance before IPA.

In order to investigate the importance and performance of various factors contributing to tourists’ overall safety perception, this research adopted a mixed quantitative approach based on Fuzzy IPA. Fuzzy IPA was adopted to determine the range of various attributes on tourists’ safety perception. The weight and logical value of safety perception were analyzed by using fuzzy analysis, and the performance and perceived service quality was analyzed by IPA. The fuzzy IPA approach is proved to be a very effective diagnostic tool for Geopark practitioners, who can use it identify current problems related to tourist’s safety perception and then assign priorities to various countermeasures.

The proposed fuzzy IPA approach which integrating fuzzy theory, Pearson correlation coefficient and importance-performance analysis, avoids mutual relationship among attributes of traditional IPA, considers the nature of fuzziness of human feeling, evaluates objectively the actual priorities of different attributes, thus provides comprehensive decision support for effective management [46]. Due to the fact that it is convenient and flexible for data collection, easy and efficient for data processing, vivid and comprehensible for understanding, fuzzy IPA approach is widely used in business analysis and marketing survey, and the computational cost of the proposed approach is less while compared with other complex processing methods such as SPSS and SAS. Thus, in this research, Fuzzy IPA approach is adopted to identify and determine the critical attributes to enhance service level and increase tourists’ satisfaction.

## Materials and methods

### Description of study area

This study was performed in the Scenic spot of the Shilin Geopark, Kunming city, located in central Yunnan Province, Southwest China. Its geographic coordinates are longitude 103°11′E to 103°29 ′E and latitude 24°38′N to 24°58 ′N [47].

It has a typical Subtropical Monsoon Climate with four distinct seasons. The annual average temperature of Shilin Geopark is 16.2°C, the highest temperature is 20.8°C in the Summer and the lowest temperature is 8.2°C in the winter [48]. Rainfall is concentrated in the July and August months, mostly in the form of thundershowers and rainstorms. The average precipitation is 967.9 mm.

Kunming Shilin (also named stone forest) National Geopark is a large-scale science park that integrates geological heritage conservation, geo-scientific research and geo-sightseeing tourism with Geological exploration [49]. The geological landscape of continental orogenic belts and subtropical forest are the main parts of this park [50]. With plenty of geological relic resources, this park is one of the most famous representatives of Karst landform in southwestern China. It is also called “The Stone Forest Museum" for it is the only place which can present the regional evolution of Karst landform in the past 25MA [50]. Abundant styles and shapes, various compositions, long time history together with unique custom of local ethnic minorities, all these brings tourists not only park beautiful scenery, but also rich cultural deposits, and thus made it one of the most famous Geopark in the world.

The Stone Forest is situated in the center of the whole scenic spot, and it consists of five areas-Major Stone Forest, Minor Stone Forest, Bushao Mountain, Liziyuanjing Scenic Area, and Perpetual Ganoderma. Most famous sights are such as Ashima, Lotus Peak, Sword Peak Pond, and Rhinoceros’ Muse upon the Moon.

### Research method and research procedure

A cross-regional survey was conducted from Aug 10^th^ to 16^th^ of 2021 in Shilin Geopark, and a questionnaire method was adopted for collecting tourists’ immediate feeling of environmental safety on the spot.

#### Establishment of environmental safety evaluation index

Referring to relevant theoretical literature about Crime Prevention through Environmental Design, together with field research of Geopark, the potential factors affecting the safety of Geoparks were summarized, and the environmental safety assessment system of Geopark is constructed [51], which is also used for investigating the tourists’ overall perception about Geopark environmental safety. The index system consists of three levels: target layer A, criterion layer B, and index layer [52, 53]. The first level is the target layer, that is, the overall environmental safety perception of tourists in Geopark, the second level is the criterion layer, including territoriality, Natural Surveillance, Access control, Activity support, image and maintenance and target hardening, these are the six elements of CPTED theory; The third level is the evaluation factor layer, namely the embodiment of the second level criterion layer, including 23 evaluation factors such as electronic monitoring facilities, public safety management, spatial boundary, spatial ownership relationship, plant configuration, lighting system, and activity facilities and so on (Table 1). The level I, II and III indexes include:

$$X_{1}=(X_{11},X_{12},X_{13},X_{14})$$

$$X_{2}=(X_{21},X_{22},X_{23},X_{24},X_{25})$$

$$X_{3}=(X_{31},X_{32},X_{33},X_{34})$$

$$X_{4}=(X_{41},X_{42},X_{43})$$

$$X_{5}=(X_{51},X_{52},X_{53},X_{54})\;X_{6}=(X_{61},X_{62},X_{63})$$

**Table 1:** Description of geopark environment safety evaluation index.

Target layer	Level I index	Level II index	Level II description
Environmental Safety Evaluation index of Geopark	Territoriality(X_1_)	spatial boundary(X_11_)	The spatial boundary is clear and well-defined
		topographical design(X_12_)	Tourist can distinguish various functional area by different topographical design
		sense of ownership(X_13_)	Fences or designs can clearly define and delineates between private, semi-private and public spaces
		plant configuration(X_14_)	Plant configuration is reasonable and tourists cannot be obscured by these plants.
	Natural Surveillance(X_2_)	electronic monitoring device(X_21_)	Enough electronic monitoring device have been installed for Surveillance
		security guard(X_22_)	Tourist’ perception of security guard service
		security management(X_23_)	Tourist’ perception of Security Administration and security patrols
		unobstructed view(X_24_)	Tourist’ perception of visibility
		interpersonal Surveillance(X_25_)	Enough surrounded crowd so that potential infringement could be reduced
	Access control(X_3_)	Entrance design(X_31_)	The design of the entrance space is reasonable and clearly identification
		Road layout(X_32_)	The roads are designed properly and connection nets are of rationality
		Signpost(X_33_)	The design and layout of indicator plays an important role
		surrounding environment(X_34_)	The surrounding environment of accesses are quite clear and easily identifiable
	Activity support(X_4_)	Safety Atmosphere(X_41_)	The Geopark has safety and positive atmosphere for geo-tourism
		sense of belonging(X_42_)	Tourists have sense of belonging in the park and willing to stay longer
		Activities equipment(X_43_)	There is enough number of safe equipment
	Image and maintenance (X_5_)	environmental sanitation(X_51_)	tourists’ attitude toward overall environmental image
		Public facilities(X_52_)	public safety facilities are maintained well
		Lighting system(X_53_)	Lighting system works well especially in the nighttime
		Uncivilized symbol(X_54_)	There is no malicious graffiti phenomenon
	target hardening (X_6_)	Safety bulletin boards (X_61_)	There are enough safety bulletin boards especially at the sites of crowded tourists
		Park service center (X_62_)	Tourists’ attitude toward Geopark service center
		Parking lot (X_63_)	Tourists’ attitude toward parking lot service

#### Questionnaire design

For convenience completed, the respondents were required to tick off the proper descriptions. In terms of content, the questionnaire consists of into two parts: a) the first part aims to sort the index factors in priority ranking order, and the research populations are the graduate students majoring in Environmental engineering and tourism management. Based on the six principles of CPTED, the respondents were asked to rank the priority of 23 factors influencing Geopark safety perception. An initial environmental safety assessment index framework was constructed at this stage. b) The second part was to collect tourists’ safety perception about Shilin Geopark. The respondents were the tourists of Shilin Park, and their evaluation of each attributes was scored. In addition, the Five Point Likert Scale is adopted, and the evaluation set *V* = (*V*_1_, *V*_2_, *V*_3_, *V*_4_, *V*_5_) = (very satisfied, satisfied, generally satisfied, dissatisfied, very dissatisfied) = (5, 4, 3, 2, 1) is established.

#### Data source and data collection

The research populations for this study were tourists who had visited Shilin Geopark. Based on the function compartmentalization, Shilin geopark mainly consists of five parts: Bushao Mountain in the northwest, the Major and Minor Stone Forests in the center, Perpetual Ganoderma in the south, and Liziyuanjing in the east, covering an area of about 12 square kilometers [2–4]. Thus, in order to guarantee the validly and generality of data, the respondents were chosen randomly from these five areas separately. This random sampling was conducted from Aug 10^th^ to 16^th^ of 2021, the respondents were asked to complete the questionnaire and participate in on-spot interview. Moreover, Commemorative stamps were sent as gifts for encouraging actively participating in the survey. 60 questionnaires were distributed and collected in each area. Finally, a total of 300 questionnaires were collected in this survey, among which 295 were valid. It is of good credibility. The results indicated that the respondents mainly consist of young and middle-aged populations ageing from 16 to 38, accounting for 52.5%. There was little sexual difference between them, the number of female tourists was 54.37% and male tourists were 45.63%. As to their occupation, students and retirees occupied 33.12% and 19.37% in respectively. Their preferable types of tourism activities were leisure walking, sightseeing and exercise, accounting for 27.50%, 23.12% and 21.88%, respectively.

#### Data processing method based on fuzzy IPA

Tourists’ safety evaluation of Geopark rise from the tourists’ psychological perception and feeling, with the characteristic of fuzziness, which can hardly be quantitatively described. Fuzzy IPA comprehensive evaluation method is an effective evaluation method based on fuzzy mathematics. It has some advantages in processing uncertainty, subjective and incomplete information [54]. Thus, it was adopted in this study for evaluating individual tourists’ subjective perception of Geopark environmental safety issue. This method combines fuzzy mathematics theory with statistical importance-performance analysis [55, 56]. The concrete steps are as follows:

Step 1: define the overall index factor set U and the weight of each Level I factor, e.g. *U* = (*U*_1_, *U*2, *U*_3_, *U*_4_, *U*_5_, *U*_6_)
Step 2: define the level II index factor set U_i_ and also their weights [57], e.g. *U*_1_ = (*U*_11_, *U*_12_, *U*_13_, *U*_14_).
Step 3: define the performance evaluation sub-factor set V
Step 4: establish the membership function of factors, and establish the comprehensive evaluation matrix R, then the membership degree and R were obtained [58, 59].
$$R=\left(\begin{array}{c} r_{11}\\ r_{21}\\ ‥\\ r_{n1} \end{array}\begin{array}{c} r_{12}\\ r_{22}\\ r_{n2} \end{array}\begin{array}{c} ‥\\ ‥\\ ‥\\ ‥ \end{array}\begin{array}{c} r_{1n}\\ r_{2n}\\ r_{nn} \end{array}\right)$$
Step 5: Calculate the fuzzy evaluation score. Fuzzy comprehensive evaluation set B is obtained based on the comprehensive evaluation matrix R.
B=W×R (1)
  Deblurring calculation, that is to say, the comprehensive evaluation score E is obtained by multiplying Fuzzy comprehensive evaluation set B by the measurement scale H [60]:
E=B×H (2)
Step 6: Compare and evaluate the scores.
Step 7: Calculate Pearson correlation coefficient, and describe correlation analysis between index factor and overall perception.
Step 8: Classification of Index Factors According to the Importance- Performance.

## Data analysis

### Reliability analysis

In order to ensure the reliability and validity of the questionnaire, SPSS 23.0 was used to conduct Cronbach reliability analysis, and it was generally known that if reliability coefficient α > 0.9, it indicated that the reliability of the scale was very good. If α > 0.8, the scale is acceptable. If α > 0.7, it means that some items in the scale need to be revised. If α < 0.7, it means that some items in the scale need to be discarded [42, 43]. The reliability coefficient α of this research is 0.913, indicating that the reliability of the questionnaire is good and fit for this study.

### Weight analysis of attributes

In the past questionnaire, the weights and value of various attributes were determined by consulting relevant industry experts and respondents’ independent self-evaluation assessment [61–63]. Although experts’ evaluation is of scientific and rational value, due to the limited number of experts, the results obtained will inevitably lead to the outcome of low commonality, strong randomness and ineffective persuasion [61]. On the other hand, large-scale tourists’ opinions survey can ensure the universal of data. Nevertheless, it cannot ensure that all the collections have high reference value because many topics in the questionnaire involve relevant professional knowledge [62].

Based on above, graduate students majoring in Environmental engineering and Tourism management of Chongqing three Georges University were selected as the subjects. The underlying reason is that they have a solid knowledge foundation of relevant disciplines, together with enough populations; they are the ideal sample for pre-survey. Thus 230 questionnaires were distributed, among which 216 were valid, 93.91% is valid. Respondents selected the most important one influencing their environmental safety evaluation based on CPTED, and then calculated the weight of each attribute. For example, among the 216 visitors, 44 students consider Territoriality (X_1_) to be the most important among the six criteria, so it can be calculated that X_1_ = 44/216 = 0.203. In the same way, the weights of other criteria and their evaluation factors can be calculated (Table 2).

**Table 2:** Weights of attributes.

Target layer	Level I index	Weight	Level II index	Weight
Environmental Safety Evaluation index of Geopark	Territoriality	0.203	spatial boundary	0.330
			topographical design	0.210
			sense of ownership	0.237
			plant configuration	0.223
	Natural Surveillance	0.302	electronic monitoring device	0.319
			security guard	0.119
			security management	0.220
			unobstructed view	0.149
			interpersonal Surveillance	0.193
	Access control	0.227	Entrance design	0.247
			Road layout	0.363
			Signpost	0.214
			surrounding environment	0.176
	Activity support	0.054	Safety Atmosphere	0.380
			sense of belonging	0.356
			Activities equipment	0.264
	image and maintenance	0.119	environmental sanitation	0.241
			Public facilities	0.203
			Lighting system	0.400
			Uncivilized symbol	0.156
	target hardening	0.095	Safety bulletin boards	0.220
			Park service center	0.325
			Parking lot	0.455

### Fuzzy evaluation analysis of tourists’ perception

Firstly, the performance set *V* = (*V*_1_, *V*_2_, *V*_3_, *V*_4_, *V*_5_) = (very satisfied, satisfied, generally satisfied, dissatisfied, very dissatisfied) = (5,4,3,2,1) is established; the evaluation index set U for environmental safety assessment of Geopark includes six level I indexes: Territoriality, Natural Surveillance, Access control, Activity support, image and maintenance and target hardening, so U = (i = 1,2,3,4,5,6), where each *U*_*i*_ consists of several level II indexes *U*_*ij*_, namely U = *U*_*ij*_.

According to Table 3, the weights of each factor are presented in the followings:

$${\text{W}}_{0}=\left(0.203,\;0.302,\;0.227,\;0.054,\;0.119,\;0.095\right)$$

$${\text{W}}_{1}=\left(0.330,\;0.210,\;0.237,\;0.223\right)$$

$${\text{W}}_{2}=\left(0.319,\;0.119,\;0.220,\;0.149,\;0.193\right)$$

$${\text{W}}_{3}=\left(0.247,\;0.363,\;0.214,\;0.176\right)$$

$${\text{W}}_{4}=\left(0.380,\;0.356,\;0.264\right)$$

$${\text{W}}_{5}=\left(0.241,\;0.203,\;0.400,\;0.156\right)$$

$${\text{W}}_{6}=\left(0.220,\;0.325,\;0.455\right)$$

**Table 3:** Performance score of Shilin Geopark environmental safety assessment.

Target layer	Level I index	Score	Level II index	Score
Environmental Safety Evaluation index of Geopark	Territoriality	3.786	spatial boundary	3.873
			topographical design	3.430
			sense of ownership	3.754
			plant configuration	4.023
	Natural Surveillance	3.798	electronic monitoring device	4.290
			security guard	3.108
			security management	4.176
			unobstructed view	3.542
			interpersonal Surveillance	3.172
	Access control	3.626	Entrance design	3.371
			Road layout	4.040
			Signpost	3.569
			surrounding environment	3.227
	Activity support	3.420	Safety Atmosphere	3.432
			sense of belonging	3.850
			Activities equipment	2.821
	image and maintenance	4.022	environmental sanitation	4.023
			Public facilities	3.800
			Lighting system	4.344
			Uncivilized symbol	3.480
	target hardening	3.262	Safety bulletin boards	3.474
			Park service center	2.711
			Parking lot	3.552

Secondly, based on the result of questionnaire survey, the ratio of respondents with the total participants in terms of each *index U*_*ij*_ is obtained, that is, R_i_(i = 1,2,3,4,5,6), the evaluation matrix of level II are listed in the following:

$$R_{1}=\left(\begin{array}{ccccc} 0.295 & 0.416 & 0.165 & 0.115 & 0.009\\ 0.195 & 0.354 & 0.215 & 0.158 & 0.078\\ 0.262 & 0.402 & 0.192 & 0.116 & 0.028\\ 0.312 & 0.461 & 0.165 & 0.062 & 0.000 \end{array}\right)$$

$$R_{2}=\left(\begin{array}{ccccc} 0.501 & 0.321 & 0.145 & 0.033 & 0.000\\ 0.151 & 0.290 & 0.225 & 0.184 & 0.150\\ 0.392 & 0.425 & 0.150 & 0.033 & 0.000\\ 0.212 & 0.358 & 0.242 & 0.136 & 0.052\\ 0.124 & 0.256 & 0.378 & 0.152 & 0.090 \end{array}\right)$$

$$R_{3}=\left(\begin{array}{ccccc} 0.156 & 0.358 & 0.254 & 0.165 & 0.067\\ 0.364 & 0.408 & 0.152 & 0.056 & 0.020\\ 0.188 & 0.385 & 0.268 & 0.126 & 0.033\\ 0.126 & 0.264 & 0.338 & 0.245 & 0.037 \end{array}\right)$$

$$R_{4}=\left(\begin{array}{ccccc} 0.186 & 0.386 & 0.188 & 0.154 & 0.086\\ 0.305 & 0.384 & 0.201 & 0.076 & 0.034\\ 0.086 & 0.165 & 0.374 & 0.234 & 0.141 \end{array}\right)$$

$$R_{5}=\left(\begin{array}{ccccc} 0.325 & 0.425 & 0.198 & 0.052 & 0.000\\ 0.258 & 0.432 & 0.196 & 0.080 & 0.034\\ 0.486 & 0.375 & 0.136 & 0.003 & 0.000\\ 0.212 & 0.348 & 0.238 & 0.112 & 0.090 \end{array}\right)$$

$$R_{6}=\left(\begin{array}{ccccc} 0.186 & 0.312 & 0.346 & 0.102 & 0.054\\ 0.053 & 0.165 & 0.354 & 0.296 & 0.132\\ 0.248 & 0.348 & 0.196 & 0.124 & 0.084 \end{array}\right)$$

According to Eq (1) and the index weights, fuzzy comprehensive evaluation set B is obtained through multiplying weight index W by comprehensive evaluation matrix R:

$${\text{B}}_{1}={\text{W}}_{1}\times {\text{R}}_{1}=\left(0.270,\;0.410,\;0.182,\;0.112,\;0.026\right)$$

$${\text{B}}_{2}={\text{W}}_{2}\times {\text{R}}_{2}=\left(0.320,\;0.333,\;0.215,0.089,0.043\right)$$

$${\text{B}}_{3}{\text{=W}}_{3}\times {\text{R}}_{3}=\left(0.233,\;0.365,0.234,0.131,0.037\right)$$

$${\text{B}}_{4}={\text{W}}_{4}\times {\text{R}}_{4}=\left(0.202,0.327,0.242,0.147,0.082\right)$$

$${\text{B}}_{5}={\text{W}}_{5}\times {\text{R}}_{5}=\left(0.358,0.395,0.179,0.047,0.021\right)$$

$${\text{B}}_{6}={\text{W}}_{6}\times {\text{R}}_{6}=\left(0.171,0.281,0.280,0.175,0.093\right)$$

The deblurring operation is done for the evaluation set at each criterion layer ac-cording to Eq (2), and then the evaluation values of level II indexes are obtained:

$${\text{E}}_{\text{1}}={5\text{B}}_{11}+{4\text{B}}_{12}+{3\text{B}}_{13}+{2\text{B}}_{14}+{\text{B}}_{15}=3.786$$

$${\text{E}}_{2}={5\text{B}}_{21}+{4\text{B}}_{22}+{3\text{B}}_{23}+{2\text{B}}_{24}+{\text{B}}_{25}=3.798$$

$${\text{E}}_{3}={5\text{B}}_{31}+{4\text{B}}_{32}+{3\text{B}}_{33}+{2\text{B}}_{34}+{\text{B}}_{35}=3.626$$

$${\text{E}}_{4}={5\text{B}}_{41}+{4\text{B}}_{42}+{3\text{B}}_{43}+{2\text{B}}_{44}+{\text{B}}_{45}=3.420$$

$${\text{E}}_{5}={5\text{B}}_{51}+{4\text{B}}_{52}+{3\text{B}}_{53}+{2\text{B}}_{54}+{\text{B}}_{55}=4.022$$

$${\text{E}}_{6}={5\text{B}}_{61}+{4\text{B}}_{62}+{3\text{B}}_{63}+{2\text{B}}_{64}+{\text{B}}_{65}=3.262$$

The final evaluation set for the Environmental assessment level is obtained through the fuzzy comprehensive evaluation method:

A=W×B=(0.274,0.358,0.216,0.110,0.042)

The deblurring operation is implemented for the final evaluation set, and the comprehensive evaluation of tourists’ perception of performance is obtained:

E=5×0.274+4×0.358+3×0.216+2×0.110+0.042=3.712

To sum up, the scoring results of the evaluation indexes for Shilin Geopark are listed in Table 3.

### Importance-performance matrix analysis

#### Pearson correlation coefficient

The Pearson correlation coefficient was used to test the influence of each factor on the overall environmental safety perception of tourists. Correlation coefficients lie between 0.8 and 1.0, indicating that the variables are very highly correlated. The correlation coefficient lies between 0.6 and 0.8 indicate that the variables are highly correlated. Similarly, Correlation coefficients lying between 0.4 and 0.6, and 0.2 and 0.4 indicate that the variables are moderately correlated and low-level of correlation. Correlation coefficients whose magnitude is less than 0.2 have little if any (linear) correlation [64].

The statistical results show that the correlation coefficient (r) between overall safety performance and electronic monitoring device, Road layout, security management and Lighting system are 0.688, 0.659, 0.635 and 0.612 respectively. These factors could be considered as highly correlated with tourists’ safety perception. Moreover, the correlation coefficient (r) between overall safety performance and uncivilized symbol, Active Atmosphere, plant configuration lies below 0.4, indicating that they are lowly correlated with overall perception. The rest are considered moderately correlated. The details can be seen in the following Table 4.

**Table 4:** The correlation analysis between index factor and overall perception.

CPTED factor	Pearson a
Overall perception	0.715**
electronic monitoring device	0.688**
Road layout	0.659**
security management	0.635**
Lighting system	0.612**
spatial boundary	0.596**
Signpost	0.585**
unobstructed view	0.583**
interpersonal surveillance	0.571**
surrounding environment	0.557**
sense of ownership	0.549**
parking lot	0.525**
Park service center	0.506**
Entrance design	0.493**
security guard	0.490**
Safety bulletin boards	0.486**
environmental sanitation	0.477**
topographical design	0.468**
Sense of belonging	0.457**
Public facilities	0.436**
Activities equipment	0.415**
Uncivilized symbol	0.349**
Active Atmosphere	0.255**
plant configuration	0.164*

^1^ * and ** respectively indicated significant at 5% and 1% level.

#### Classification of index factors according to the importance-performance

**Fig 1:**
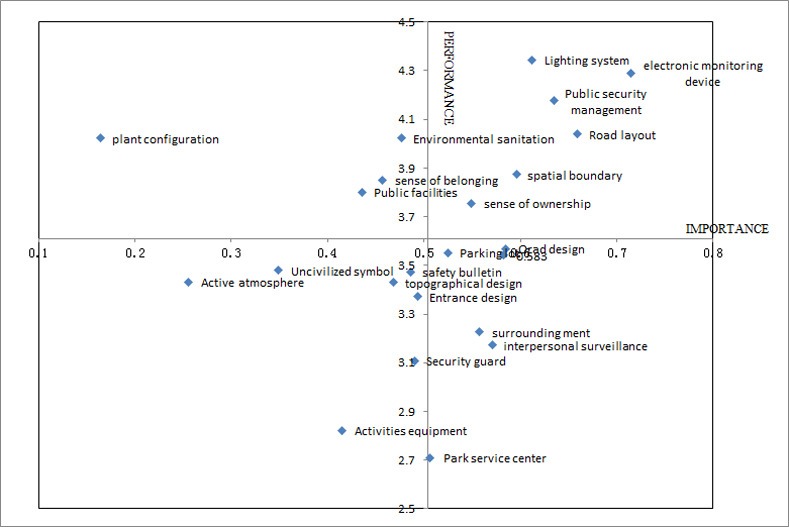
Importance-performance matrix of attributes.

The importance degree of index factors was taken as the abscissa and the environmental safety performance degree of tourists as the ordinate [64]. Then all these 23 factors were classified in the following 2×2 importance-performance matrix, which can be seen in Fig 1.

## Results

### Fuzzy comprehensive evaluation result

As shown in Table 3, the overall safety evaluation performance of Shilin Geopark was 3.712, it lies between 3 and 4, which means that the tourists were general satisfied with environmental safety design of Shilin Geopark.

The score of territoriality was 3.786; it signified that tourist was generally satisfied. Among Level II factors, the performance score of spatial boundary and plant configuration were 3.873 and 4.023, respectively. Within Shilin park, stone forests of various geology characteristic are distributed in different areas, and the Spatial boundary line between each is clear. These could provide tourists’ a strong sense of spatial belonging [65]. In terms of plant configuration, the overall arrangement of plants in Shilin Geopark is reasonable, and the trees, shrubs and grasses are well matched and planted. The score of topographical design is 3.430. Based on on-spot interview and observation, most Sightseeing sites are strewn at random in Shilin park, and the terrain is higher in the north and lower in the south, which is called defendable space. This kind of design is beneficial for effectively space division and internal natural Surveillance, which at last enhance tourists’ feeling of security [66]. The score of sense of ownership is 3.754, indicating a clear sense of belonging among tourists. In particular, the 40-meter high Rock pillar and Ashimar sculpture stands independently in the center of major and Minor stone forest, acts as the landmark structure of each area, increase tourists’ sense of identity and enhance their feeling of safety.

Tourists’ perception score about Natural Surveillance was 3.798, signifying generally satisfied with this. Among sub-factors, the performance score of the electronic monitoring device, security guard and Public security management were 4.290, 3.108 and 4.176, respectively. These scores indicated that artificial monitoring equipment played a very important role in the crime prevention of Shilin Geopark. In fact, the wide coverage of electronic monitoring within the whole park is fully considered by the Shilin administrators, and sufficient patrol personnel are also recruited for safety consideration. However, the service of security guide should be strengthened based on the survey. In subsequent, the score of unobstructed view was 3.542, which indicated that natural condition factor, including un-obstructed view and space configuration all had contributed to the enhancement of the safety perception of tourists. The score of interpersonal surveillance is 3.172, indicating that the surveillance from the surrounding people enhances the safety confidence of tourists to some extent. However, Due to the fact of large coverage and uneven terrain, coupled with the obstruction of the natural landscape of Stone forest, the road accessibility of timely aid is relatively low, these finally led to low score of personal surveillance [67, 68]. For example, Bushao Mountain scenic spot is enclosed by clear boundaries and has clear spatial ownership. However, the high wall blocks the line of sight and hinders the monitoring from the outside. In case of danger, it is impossible to get timely rescue from the surrounding population. In addition, the densely enclosed plant in many sites also hinders effective interpersonal surveillance from the surrounding populations, thus lowers tourists’ feeling of safety [68].

Tourists’ safety perception of access control was 3.62. As to geographical location, Shilin Geopark is located in the extension of the city center, it is just 78 km far from the provincial capital Kunming. This provides convenient transportation and strong accessibility for it [69]. Among sub-factors, the score of entrance design is 3.371; it shows that tourists felt moderate satisfaction about its safety design, in fact, there are 9 entrances of the park, which are located in different parts, it inevitable increase the difficulty of public security management [70], thus reduced the security consideration of tourists. The score of road layout is 4.040, indicating that the road network design of the internal space is perceived as satisfied. However, it is necessary to mention that, due to the coverage of more than 400 square kilometers and hundreds of sight-seeing sites, the roads of each area cannot form loops, which will reduce the tourists’ space safety recognition to some extent [71]. For example, Penglai island located in the center of the stone forest long lake. With only one long walking path connecting it with other islands, it became an "isolated island in the lake". Therefore, attention should be paid to the connectivity and integration of the road design. The perception scores of the Signpost is 3.569, indicating that the easily identifiable Signpost in the park increases the sense of safety. The perception score of the surrounding environment is 3.227, which is relatively low in this index system; As to the reasons, Shilin Park is located in the countryside, with relatively limited public safety resources like first aid and monitoring resources, this is not beneficial for promoting tourists’ safety feeling.

Tourists’ safety perception of Activity support was 3.380, in fact, series of cultural and Geographic sightseeing entitles a positive atmosphere of tourism [72]; activities held in Shilin park includes the geographical exhibition, exercise, cultural performance and so on. Among them, the score of Safety Atmosphere and sense of belonging were 3.432 and 3.850, respectively, indicating that stable park atmosphere can make users feel safe and comfortable. For example, the Liziyuanqing Scenic Area has a wide view and beautiful scenery. In the morning and evening, many residents do exercise there, coloring the active atmosphere of the park, and virtually enhance peoples’ feeling of safety. While on the other hand, the score of "Activities equipment" is only 2.821, which is relatively lower than the average score. Through interviews, it is found that lacking enough facilities for tourists’ rest, together with increasing number of tourist’s crowd into the park, these reduce tourists’ perception of safety.

Tourists’ safety perception of image and maintenance was 4.022, among which the score of environmental sanitation was 4.023. It showed that this park was good at maintaining a positive image and promoting safety communication. Taking Long lake as an example, it is dominated by waterfront landscape. With open space, good infrastructure and sanitary conditions, this site has many brightly colored leaf trees planted along the road beside the lake. The beautiful scenery has attracted thousands of tourists visiting here and taking photos at these sites. Moreover, the score of public facilities and uncivilized symbols were 3.800 and 3.480 respectively. Data shows that the park pays attention to the maintenance of park image in general, and has recruited personnel team for environmental maintaining. However, some improvements need to be done in the Waterfall cave and Millennium Yushu areas of Perpetual Ganoderma, due to their poor accessibility, together with lacking of maintenance of vegetation and facilities, these sites finally resulting in a negative environmental atmosphere, which is not beneficial for tourists’ safety. on the contrary, the overall score of lighting system is 4.344, indicating that the light coverage of Shilin Geopark is wide and the visibility is strong, this increases tourists’ safety perception especially in the dark carve areas.

Tourists’ Perception with target Targeted enhancement was 3.262, which is the lowest score. Among them, the score of Park service center is only 2.711, lower than the average score. The reasons underlying it were that there were relatively limited facilities for catering and rest, which cannot meet the increasing demand of tourists’ leisure and relaxation needs. The performance score of Safety bulletin boards is 3.474. Although there are enough Safety bulletin boards in the park to remind visitors to watch out. However, through the investigation, results show that the distribution of safety bulletin boards in the park is un-reasonable, and many potentially dangerous areas, such as construction areas and deep pools, have not installed corresponding warning signs, which is not conducive to the safety of tourists in the park. Lastly, the performance score of the parking lot is 3.552, indicating that the area planning and layout of the parking area are reasonable. However, through the interview, it was found that some burglary cases often occurred in the parking area. Therefore, a scientific and reasonable way should be taken to reduce the probability of criminals committing in the parking area, appropriate human intervention should be taken to protect the safety of tourists’ personal and property safety [73].

### Importance-performance analysis

Seen from Fig 1, all the attributes are classified into four quadrants based on IPA.

The first quadrant (advantage region) contains the factors of high importance and high performance, these issues include electronic monitoring device, Lighting system, Public security management, Road layout and etc., These are the most important attributes in tourist’ opinion and they are also considered as strengths of Shilin geopark, in fact, with the rapid development of Chinese geo-tourism, Geopark practitioners have increased the investment of hardware, including electronic monitoring device, Lighting system, Road layout and so on. Therefore, tourists attach great importance on these factors and are very satisfied with them. These aspects should be maintained and enhanced in the future.

The second quadrant (maintaining region) describes the factors of lowly importance and high performance, these issues include environmental sanitation, sense of belonging and Public facilities, this category indicated that although tourists are generally satisfied with these, too much resource should not be invested on them for they have low weights in tourist opinion.

The third quadrant (opportunity region) described the factors of lowly importance and lowly performance, these issues include Active Atmosphere, Uncivilized symbol, topographical design, Entrance design, etc., these are the weakness of Shilin Park, and tourists paid no attention on these, therefore geopark may notice this and make some improvements.

The fourth quadrant (improvement region) described the factors of high importance and lowly performance, these issues include Road design, parking lot, surrounding environment, interpersonal surveillance and security guard, tourist attach great importance on these factors while their perceived quality is low, this indicates that tourists perception of ‘pay-value in return’ is low. These are the main weakness of Shilin park, and geopark needs sustainable improvement in these aspects for attracting more tourists, for the improvement of these factors will raise tourists’ performance significantly.

## Discussion, conclusions and implications

### Discussion and conclusions

Based on the analysis above, it can be seen that the overall Environmental Safety Evaluation score was 3.712, signifying that tourists were generally satisfied. In specific, the score of image and maintenance was the highest one, activity support and the target hardening were the lowest one. This is consistent with [7, 12], which stated that compared with software and management improvement, Chinese parks focused more on the hardware input and physical equipment input. In fact, numerous studies have pointed out that the inner management competence and service level are the key factors contributing to tourists’ perception of safety as well as satisfaction.

Furthermore, among all the 23 level-two factors, the score of 21 factors lied between 3 and 5, it means that the environmental design and maintenance generally met the safety demands of tourists. However, two factors are still less than 3, they are Activities equipment and Park service center, thus greater attention should be paid on these improvements.

Through the Pearson correlation coefficient analysis, the factors influencing tourists’ Safety Evaluation are ranked from highest to lowest, and the top 5 ones are: electronic monitoring device, Road layout, Public safety management, Lighting system and spatial boundary. Among these five ones, electronic monitoring device, Lighting system, Road layout, Public safety management and road layout are of high importance and high performance. This result is quite consistent with [14] and [38], which stated that some security precautions including Lighting system, interpersonal surveillance and Security guards patrol were playing key role for enhancing tourists’ safety confidence. However, the role of road layout had been neglected by previous researchers which mainly focused on CPTED at neighborhood areas and urban area, thus this research pointed out that long-view distance road design is also critically important. This was also consistent with [39]. Although with low score of performance, the importance of physical boundaries should not be neglected, it ranked 5^th^ in the priority order of Pearson Correlation Coefficient. In fact, this result supports the results of [63] and [65], and they also argued that Physical boundaries, such as the walls, fences and plants between two areas may limit the possibilities for potential offenders’ escape, and carefully design of evacuation passageway and safety exit, will enhance tourists’ safety confidence.

In subsequent, the factors including Signpost, unobstructed view, interpersonal surveillance, as well as surrounding environment play relatively important role for tourists’ safety perception. And the least important factors include plant configuration, Active Atmosphere and Uncivilized symbol. This is not consistent with [14, 34], which emphases the importance role of vegetation maintenance and legitimate activities. The underlying reason behind it may attribute to the nature of Geopark, there are huge amount of stones compared with plants, furthermore the open space for conducting legitimate activities is also small, thus their importance is considered as relatively less.

While on the other hand, from the aspects of importance analysis, signpost, unobstructed view and interpersonal surveillance, surrounding environment all have played an important role for tourists’ overall safety perception; this is consistent with [28, 30]. They all emphasized the increasement the Visibility of the crime targets and reduce the probability of being violating through unobstructed view and interpersonal surveillance. However, tourists’ perception of performance level of these factors is relatively low in Shilin geopark, so attention should be paid on these shortcomings, and feasible plans should be made to reduce potential safety hazard in these areas.

### Policy implications

#### Optimize the layout and design of park spaces

Based on the analysis above, results showed that a good layout can enhance tourists’ sense of belonging and increase their feeling of safety, On the contrary, the chaotic layout of the space is inclined to make tourists’ confused and discomfort [74]. Therefore, good planning and designing of inner space are necessary, in the process of optimization, attentions should be paid on the following: a) spatial boundary is the foundation of Road layout, Entrance design, surrounding environment as well as a sense of ownership. Thus the boundary line of each areas should be clear and definitely; b) In terms of plant configuration, good visibility should be guaranteed, the purpose is to reduce the possibility of "criminal blind area" sheltered by plants [75]; c) In terms of terrain design, measures should be taken to make full use of the existing geographical resources to broaden activity place. At the same time, thatched pavilions are needed in each area for providing shady retreats for relaxing; d) As to the path design, the continuity and accessibility of the road network should be strengthened to avoid the occurrence of the dead-end road; e) In the lighting system, the main road and activity space should be illuminated day and night, in case of emergency [76].

#### Strengthen the natural and interpersonal surveillance

As to the inner management, electronic monitoring facilities should be installed at each site, especially in remote sites with low pedestrian volume [77, 78]. At the same time, the inspection of the strangers at the entrance should be strengthened, and the management of public security should also be enhanced. Finally, enough safeguards and Patrol personnel should also be recruited for optimizing the park safety system.

#### Consolidate the image of environmental safety

Environmental image provides tourists with the most intuitive visual impression and safety feelings [79]. A Park with a comfortable environmental image can not only bring tourists spiritual pleasure, but also encourage them to stay longer, and these inevitably results in an effective interpersonal surveillance [80]. Secondly, some interesting facilities like Rock climbing, Giant Swing and Children’s Bouncy Castles are also needed for attracting tourists to participate in group programs, thus reduce their feeling of loneness and insecurity. Thirdly, professional safety persons should be recruited to monitor and maintain the safety equipment and facilities regularly, thus to consolidate the image of environmental safety.

## Supporting information

S1 Data Descriptive statistics. (XLSX)

S2 Data IPA description. (XLS)
